# Multi‐omics analysis reveals BZW1's regulation of EMT via the Wnt pathway in lung adenocarcinoma

**DOI:** 10.1111/jcmm.70163

**Published:** 2024-10-27

**Authors:** Wei Lai, Zhou Ping, Yun Chen, Junrong Wang, Yuyan Liu, Shishi Zou, Jieweng Wang, Tianyu Zhang, Wei Ren, Wei Wang

**Affiliations:** ^1^ Department of Thoracic Surgery Renmin Hospital of Wuhan University Wuhan China; ^2^ Wuhan Children's Hospital (Wuhan Maternal and Child Healthcare Hospital) Tongji Medical College Huazhong University of Science & Technology Wuhan China; ^3^ Department of Thoracic Surgery Xishui People's Hospital Affiliated to Hubei University of Science and Technology Huanggang Hubei China; ^4^ Department of Head and Neck Surgery Harbin Medical University Cancer Hospital Harbin China; ^5^ Department of Cardiac Surgery Renmin Hospital of Wuhan University Wuhan China

**Keywords:** BZW1, EMT, lung adenocarcinoma, Wnt/β‐catenin

## Abstract

Exploring the role of novel cancer gene BZW1 in lung adenocarcinoma (LUAD) and unveiling associated signalling pathways. Firstly, we conducted a pan‐cancer analysis of BZW1 using multiple databases. Subsequently, leveraging single‐cell data from LUAD, we successfully uncovered potential oncological processes associated with BZW1 and further validated them through experimentation. Simultaneously, we continued to investigate the potential molecular mechanisms underlying the oncological processes mediated by BZW1. Additionally, we employed various machine learning algorithms to construct prognostic models concerning BZW1 and the epithelial‐mesenchymal transition (EMT) process. Our research firstly demonstrated the elevated expression of BZW1 in various cancer cells. Leveraging single‐cell data from LUAD, we identified that BZW1 regulates the occurrence of EMT in LUAD, a phenomenon validated across multiple LUAD cell lines. Moreover, we further discovered that BZW1 regulates LUAD's EMT process through the Wnt/β‐catenin signalling pathway. Lastly, we successfully constructed prognostic models using BZW1‐related genes and EMT genes.

## 1 INTRODUCTION

To date, targeted therapy has become one of the preferred methods for treating most cancers.^1^ However, resistance often occurs during targeted therapy.^2^ This may be due to the emergence of resistant mutations, compensatory activation of alternative signalling pathways, or the preexistence of cells with dominant growth‐carrying resistance gene alterations. Lung cancer is a global healthcare burden and targeted therapy has become one of the preferred methods for its treatment.^3^ Over the past few decades, clinical studies have shown that multi‐targeted therapy is significantly more effective than single‐targeted therapy. Therefore, exploring more treatment targets for lung cancer is of great significance.

Basic leucine zipper and W2 domains 1 (BZW1), also known as 5MP2, BZAP45 and Nbla10236, is a member of the basic leucine zipper (bZIP) superfamily of transcription factors. The BZW1 gene encodes a 45 kDa protein containing an N‐terminal bZIP domain for protein interactions and a C‐terminal nucleotide (ATP or GTP) binding domain.^4^ Previous studies have demonstrated that human BZW1 can activate transcription of the histone H4 gene and serve as a co‐regulator with other transcription factors to control the cell cycle. In recent years, BZW1 has been identified as enhancing phosphorylation to promote glycolysis in pancreatic ductal adenocarcinoma.^5^ Moreover, BZW1 has been found to regulate the cell cycle in ovarian cancer, thereby promoting its progression.^6^ Additionally, BZW1 plays a crucial role in mucoepidermoid carcinoma of the salivary glands.^7^ However, little is known about the role of BZW1 in lung adenocarcinoma (LUAD). The transition of tumour cells from an epithelial to a mesenchymal state, known as epithelial‐mesenchymal transition (EMT), is a crucial driver of tumour invasion.^8^ EMT processes have been reported in most tumours, suggesting the potential efficacy of inhibiting EMT as a therapeutic strategy for LUAD. Among the myriad cellular pathways influencing EMT, the Wnt/β‐catenin pathway emerges as pivotal.^9^ The Wnt signalling cascade is intricately linked to numerous biochemical processes, including cancer cell proliferation, apoptosis and autophagy.^9^ Mutations in the Wnt pathway can precipitate various pathological conditions associated with abnormal growth and cancer progression.^10^ β‐catenin, as a functional effector molecule of the Wnt signal, undergoes critical modifications and degradation events within this pathway.^11^ Dysregulated activation or suppression of this pathway correlates closely with EMT progression. Wnt signalling inhibits the expression of glycogen synthase kinase‐3β (GSK‐3β) by binding to receptors on the cell membrane, thereby impeding β‐catenin degradation and promoting its nuclear translocation. This activation of the Wnt/β‐catenin pathway induces downstream target genes, ultimately facilitating EMT in tumour cells.^12^ Numerous studies have established a significant association between EMT processes and the Wnt/β‐catenin pathway in LUAD, suggesting that blockade of the Wnt/β‐catenin pathway could attenuate tumour cell progression.

Our study initially employed bioinformatics approaches to identify a trend of high expression of BZW1 in various cancer cells. Subsequently, utilizing single‐cell sequencing data, we discovered its predominant high expression in malignant cells, epithelial cells and fibroblasts within tumour cells. Further analysis of single‐cell sequencing data validated the close association between BZW1 and the occurrence and progression of EMT. To elucidate the specific mechanisms by which BZW1 promotes EMT, we conducted additional investigations and found that BZW1 can induce EMT in tumour cells through the Wnt/β‐catenin pathway. Additionally, by intersecting BZW1‐related genes with genes involved in EMT, we successfully constructed a prognostic model using machine learning algorithms such as WGCNA and LASSON. In summary, our multi‐omics approach demonstrates that BZW1 influences the occurrence of EMT in LUAD via the Wnt/β‐catenin pathway.

## 2 MATERIALS AND METHODS

### 2.1 Pan‐cancer analysis data source

We obtained sequencing and clinical data of LUAD from The Cancer Genome Alta (TCGA) (The Cancer Genome Atlas Program (TCGA) ‐ NCI), an openly accessible data source. Utilizing the TIMER 2.0 database (https://cistrome.shinyapps.io/timer/), we analysed the role of BZW1 across 33 cancer types, supplemented by GEPIA data for cancers lacking normal or tumour samples (GEPIA (Gene Expression Profiling Interactive Analysis) (cancer‐pku.cn)).^13^, ^14^ Additionally, GEPIA provided insights into the role of BZW1 across different stages of cancer. Furthermore, we analysed the protein expression of BZW1 in breast cancer, renal clear cell carcinoma and LUAD using UALCAN (https://ualcan.path.uab.edu/analysis‐prot.html).^15^ The Human Protein Atlas (HPA) database facilitated the determination of BZW1's specific cellular localization (https://www.proteinatlas.org/).^16^

### 2.2 Sources of single‐cell data analysis

We used the CancerSEA (https://bioinfo.life.hust.edu.cn/CancerSEA/) database to analyse single‐cell data sourced from multiple datasets to explore 14 primary tumour‐associated cellular activities associated with BZW1.^17^ The correlation was visualized using ggplot2 in R language. Furthermore, to delve into the expression patterns of BZW1 across different cell types, we employed TISCH2 (https://tisch.comp‐genomics.org/) to analyse LUAD single‐cell datasets from the GEO database (https://www.ncbi.nlm.nih.gov/geo/), with subsequent annotation. Meanwhile, to elucidate BZW1's functional insights, we conducted genomic data analysis using (https://www.cbioportal.org/) to investigate the impact of mutations at different loci of BZW1 on various cancers to reveal its functional roles further.^18^

### 2.3 Analysis of immune cell infiltration

To further elucidate the relationship between BZW1 and its associated genes with immune cells, we initially employed the Timer2.0 database to analyse the correlation between BZW1‐associated genes and CD8+ T cells and CD4+ T cells. Subsequently, we conducted analyses using R language across multiple immune databases, including CIBERSORT, XCELL and EPIC, to explore the correlation between BZW1 and immune cells, presenting the results through heatmaps. Moreover, to delve deeper into the relationship between BZW1 and immune‐related checkpoints, we further utilized the R package ‘CIBERSORT’ to analyse the association between immune checkpoints and BZW1, displaying the relationship through heatmap visualization.

### 2.4 Constructing prognostic models using machine learning algorithms

Single‐cell sequencing analysis reveals a close association between BZW1 and the EMT process. To further elucidate this phenomenon, we intersected EMT genes with BZW1‐associated genes. Concurrently, we constructed a prognostic model using Weighted Gene Co‐expression Network Analysis (WGCNA) and Least Absolute Shrinkage and Selection Operator (LASSO) algorithms successfully validating its efficacy. This discovery further substantiates the intimate relationship between BZW1 and the EMT process. The risk score formula was defined as follows: risk score = (expression level of gene a × coefficient a) + (expression level of gene b × coefficient b) + … + (expression level of gene n × coefficient n).

### 2.5 Cell culture and drug stimulate

We selected two non‐small cell lung cancer cell lines (A549 and H1299), both procured from the American Type Culture Collection (ATCC) and cultured under standard conditions. A549 cells were cultured in F‐12 k medium containing 10% serum, while H1299 cells were cultured in 1640 medium containing 10% serum (All sourced from Pricella). Both cell lines were maintained in a 37°C incubator with 5% CO_2_. To further investigate the function of EMT, we supplemented the culture with TGF‐β1 (10 ng/mL) to promote EMT occurrence. To elucidate the role of the Wnt/β‐catenin pathway, we added a GSK‐3β inhibitor (LiCl) to activate Wnt pathway activation.^19^

### 2.6 Plasmid transfection and stable cell line generation

We cloned the cDNA of BZW1 into the pcDNA3.1 plasmid to generate a plasmid for transient transfection using Lipofectamine 8000 (Beyotime, Shanghai), aiming to upregulate BZW1 expression. To obtain stable BZW1 knockdown cell lines, lentiviral vectors carrying BZW1 (LV‐BZW1) or control (LV‐control) sequences were packaged. Subsequently, stable cell lines with reduced BZW1 expression were selected using puromycin. (sh‐1: GGCTTAACTGAAACCGGTACT; sh‐2: GCAGTAGCTAAGTTTCTTGAT).

### 2.7 Wound healing assay

Cells were seeded into six‐well plates and cultured for 2–3 days until reaching confluence. To minimize the effects of cell proliferation on cell migration, we cultured the cells in a low‐serum medium (<2%). Furthermore, before conducting the wound healing assay, we treated the cells with 1 μg/mL mitomycin C for 1 h to inhibit cell division. Scratch wounds were created on the cell surface using a 200 μL pipette tip, followed by washing with PBS to remove cell debris. Fresh medium was replaced simultaneously and images of the wounds were captured at 0, 24 and 48 h time points. The percentage of wound closure was calculated using the formula: Percent wound closure (%) = (initial area − final wound area)/initial area.

### 2.8 Cell viability assessed by CCK‐8 assay

Cells were digested with trypsin before and after drug treatment, suspended in culture medium and counted using a cell counting plate. Four thousand cells were seeded into each well of a 96‐well plate and cultured for 24 h. After incubation, cell viability was assessed using the CCK‐8 assay according to the manufacturer's instructions. Specifically, 10 μL of the CCK‐8 reagent (Glpbio, America) was added to each well and the plate was then incubated at 37°C for 2–3 h. Absorbance was measured at 450 nm using a microplate reader to evaluate cell viability.

### 2.9 Cell cycle

Cells collected before and after drug treatment were digested with pancreatic enzymes to obtain single‐cell suspensions, washed with PBS and resuspended after centrifugation. Then, they were fixed overnight at 4°C in pre‐chilled 75% ethanol. The following day, 400 μL of 50 μg/mL propidium iodide (PI) solution and 50 μL of 100 μg RNaseA were added, followed by a 30‐min incubation period. Subsequently, analysis was performed using a Beckman flow cytometer. All sources were purchased from Servicebio.

### 2.10 Detection of cell apoptosis

Apoptosis levels in cells following BZW1 knockdown were detected using apoptosis assay kits with FITC and PI (Elabscience, Wuhan). Control and experimental group cells were cultured under identical conditions in six‐well plates for 48 h. Following incubation, apoptosis levels were assessed using FITC and PI channels. Flow cytometry analysis was performed using a flow cytometer from Beckman Coulter, America.

### 2.11 Cell migration and invasion assay

Migration and invasion assays of tumour cells were conducted using Transwell chambers (Corning, America). Cells before and after drug treatment were digested with trypsin and suspended in 15 mL centrifuge tubes containing culture medium. Cell counting was performed using a cell counting plate and 2.5 × 10^4^ cells were added to the upper chamber of each Transwell insert. To create migration conditions, the upper chamber medium was FBS‐free, while the lower chamber contained 20% FBS. The medium volume in the upper chamber was approximately 200 μL, and in the lower chamber was approximately 800 μL. After 48 h of incubation, cells were fixed with paraformaldehyde (Servicebio, Wuhan), stained with crystal violet (Servicebio, Wuhan) and five random fields were selected under a microscope (400×, Olympus) for cell counting. For the invasion assay, cells were cultured in a Matrigel (Corning, America). The procedure involved thawing the Matrigel, followed by incubation in a cell culture incubator for 2 h for gel hydration. Subsequent steps were identical to those of the migration assay.

### 2.12 Western blot

Following the protocol previously reported by others, proteins were collected and subjected to SDS‐PAGE for separation based on their molecular weights.^20^ PMSF and phosphoprotein phosphatase inhibitors (Servicebio, Wuhan) were added according to the manufacturer's instructions. The separated protein samples were then transferred onto PVDF membranes. A rapid closure solution (Epizyme, Shanghai) was applied to minimize nonspecific binding. Primary antibodies were incubated at room temperature for 1–2 h or overnight at 4°C, while secondary antibodies were incubated at room temperature for 1–2 h. Protein signals were detected using a Bio‐Rad ChemiDox XRS+ system. The relevant antibodies used are as follows. E‐cadherin(20874‐1‐AP), N‐cadherin (66219‐1‐AP), Vimentin (660330‐1‐lg) and GAPDH (60004‐1‐lg) were procured from Proteintech. The BZW1 (A3359) and GSK‐3β(A2081) were purchased by Abclonal. The β‐catenin (WL0962a) was obtained from Wanleibio.

### 2.13 Statistical analysis

All data were represented using mean ± standard deviation (mean ± SD). Bar charts were created using GraphPad Prism 9.5.0 software, while other visualizations were generated using R software (version 4.2.1). Differences between two groups were assessed using independent samples *t*‐test and one‐way analysis of variance (ANOVA) was performed with Sidak's and Tukey's to evaluate between‐group differences. *p*‐value less than 0.05 was considered statistically significant.

## 3 RESULTS

### 3.1 Expression profile of bzw1 across 33 cancer types

**FIGURE 1:**
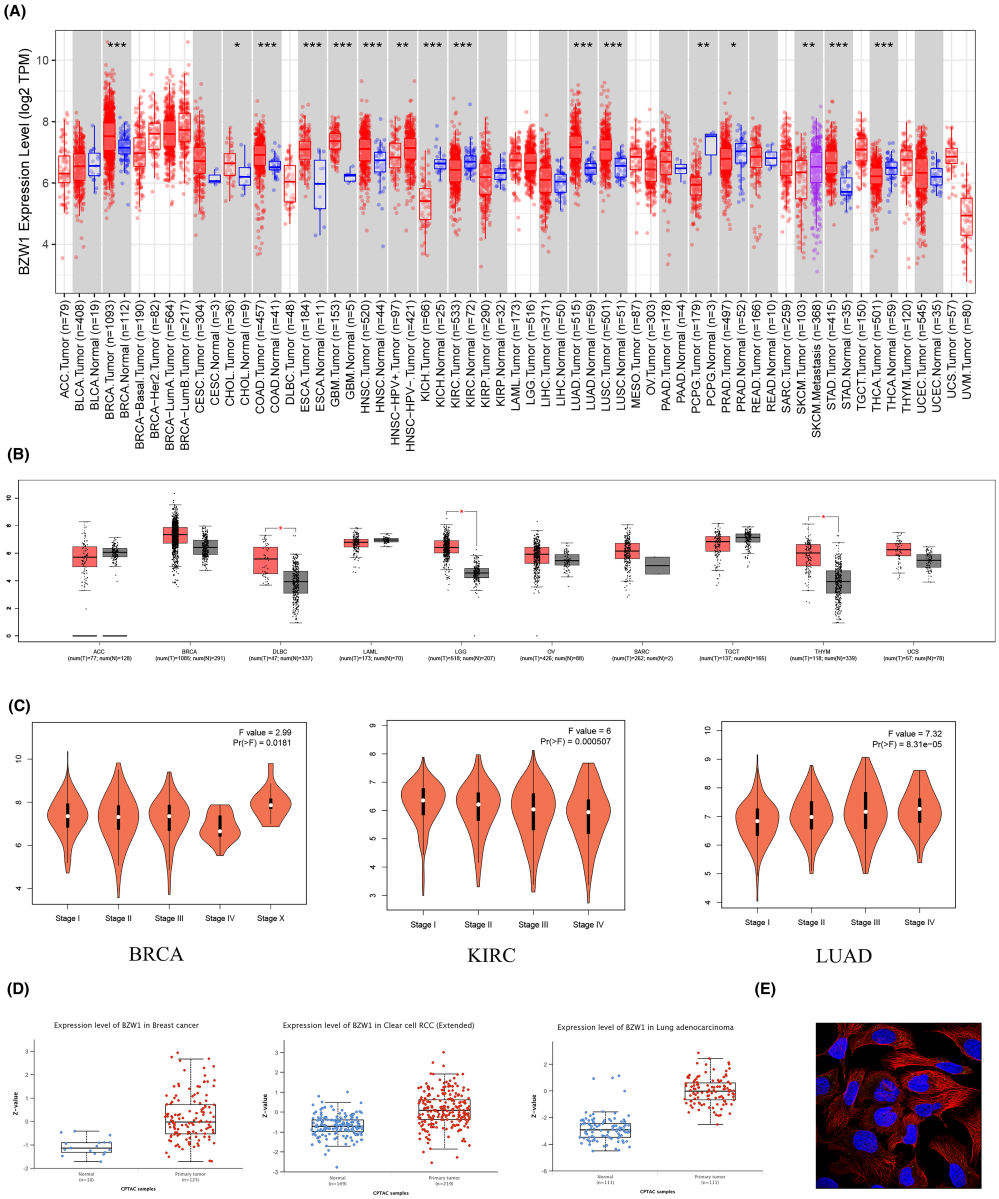
Pan‐cancer analysis of BZW1. (A) Analysis of BZW1 expression across 33 cancer types using the Timer2.0 database (https://cistrome.shinyapps.io/timer/). (B) Analysis using the GEPIA database to address cancer types lacking normal or tumour samples (GEPIA (Gene Expression Profiling Interactive Analysis) (cancer‐pku.cn)). (C) Impact analysis of BZW1 on different cancer stages. (D) Protein expression profile of BZW1 across different tumours (https://ualcan.path.uab.edu/analysis‐prot.html). (E) Subcellular localization analysis of BZW1 using the HPA database. Blue fluorescence represents the cell nucleus, red fluorescence represents the cell cytoskeleton and green fluorescence represents BZW1 (https://www.proteinatlas.org/). (*p < 0.05, **p < 0.01, ***p < 0.001).

We initially utilized the Timer2.0 database and GEO data to analyse the expression pattern of BZW1 across 33 cancer types, revealing predominantly high expression levels in most cancers (Figure 1A,B). To further explore the role of BZW1 in tumour progression, we conducted stage‐specific expression analyses focusing on cancers with significant expression differences between tumour and normal groups (Figure 1C). Our findings indicate a significant impact of BZW1 on the staging of breast cancer (BRCA), kidney renal clear cell carcinoma (KIRC) and LUAD. Additionally, we employed the UALCAN database for online analysis of BZW1 protein expression in BRCA, renal clear cell carcinoma and LUAD (Figure 1D). Furthermore, Figure 1E illustrates the subcellular localization of BZW1.

### 3.2 Genomic analysis reveals BZW1 landscape across 33 cancer types

**FIGURE 2:**
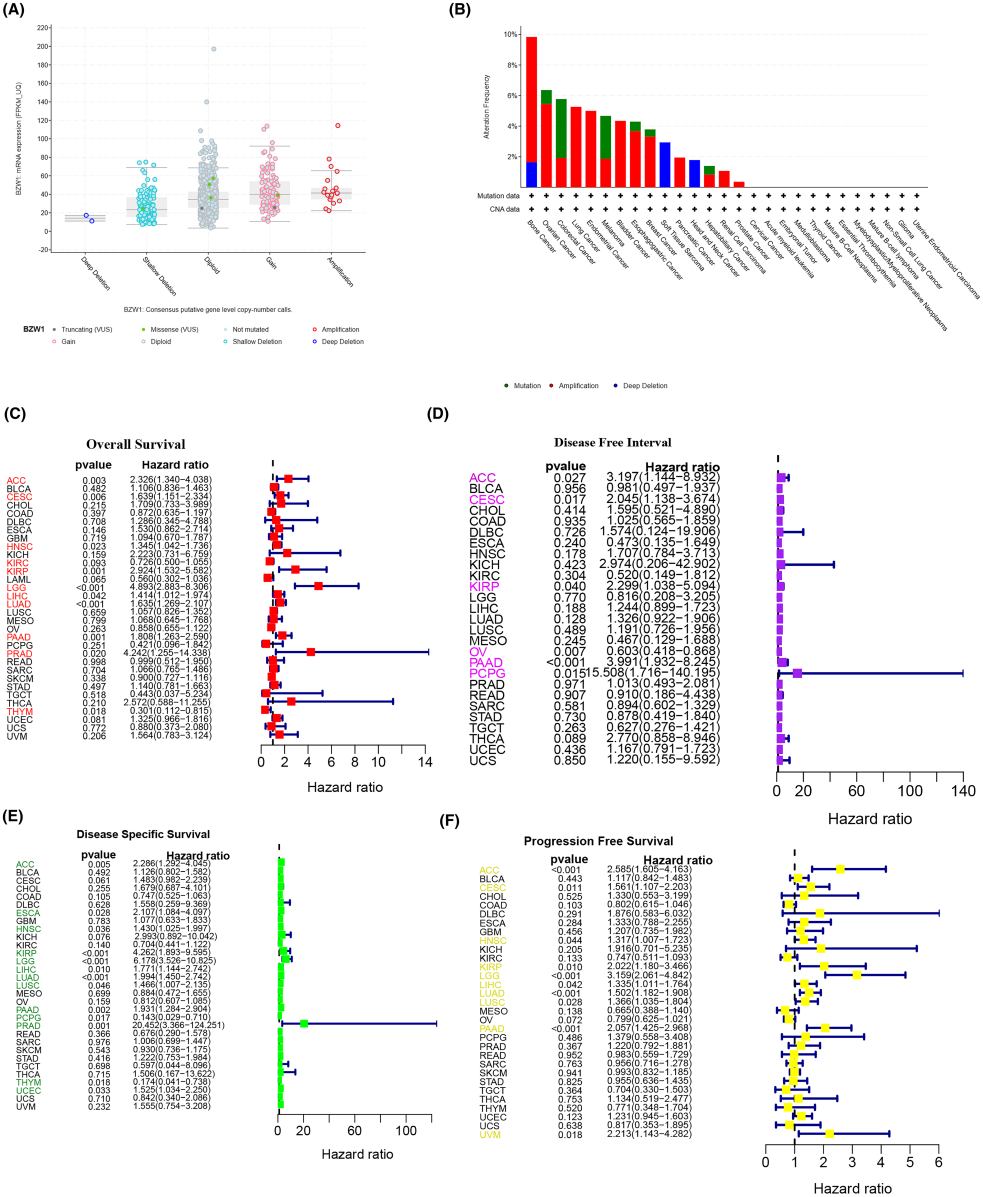
Relationship between BZW1 and Survival Rates across 33 Cancer Types. (A) Analysis of gene mutations in BZW1 using cBioPortal (https://www.cbioportal.org/). (B) Analysis of BZW1 mutations across different cancer types in TCGA, showing only significant cancer types. (C) Analysis of the relationship between BZW1 and overall survival (OS) using clinical data from TCGA. (D) Analysis of the relationship between BZW1 and disease‐free interval (DFI) using clinical data from TCGA. (E) Analysis of the relationship between BZW1 and disease‐specific survival (DSS) using clinical data from TCGA. (F) Analysis of the relationship between BZW1 and progression‐free survival (PFS) using clinical data from TCGA.

Given the impact of genetic alterations on tumour progression, we further scrutinized the mutational landscape of BZW1 across diverse cancer types (Figure 2A). Mutations in BZW1 predominantly appeared as gain‐of‐function alterations (Figure 2B). Notably, BZW1 mutations were most prevalent in bone cancer, ovarian, colorectal and lung cancers, hinting at a potential association between BZW1 mutations and specific cancer types. Figure 2C–F depict the influence of BZW1 on overall survival (OS), disease‐free interval (DFI), disease‐specific survival (DSS) and progression‐free survival (PFS), respectively. Remarkably, we observed concordance between the impact of BZW1 on survival outcomes across different cancer types and its mutational profile within cancers. This finding further underscores the significant role of the BZW1 gene in tumour progression and patient survival.

### 3.3 Single‐cell sequencing analysis reveals tumour‐associated functions of BZW1

**FIGURE 3:**
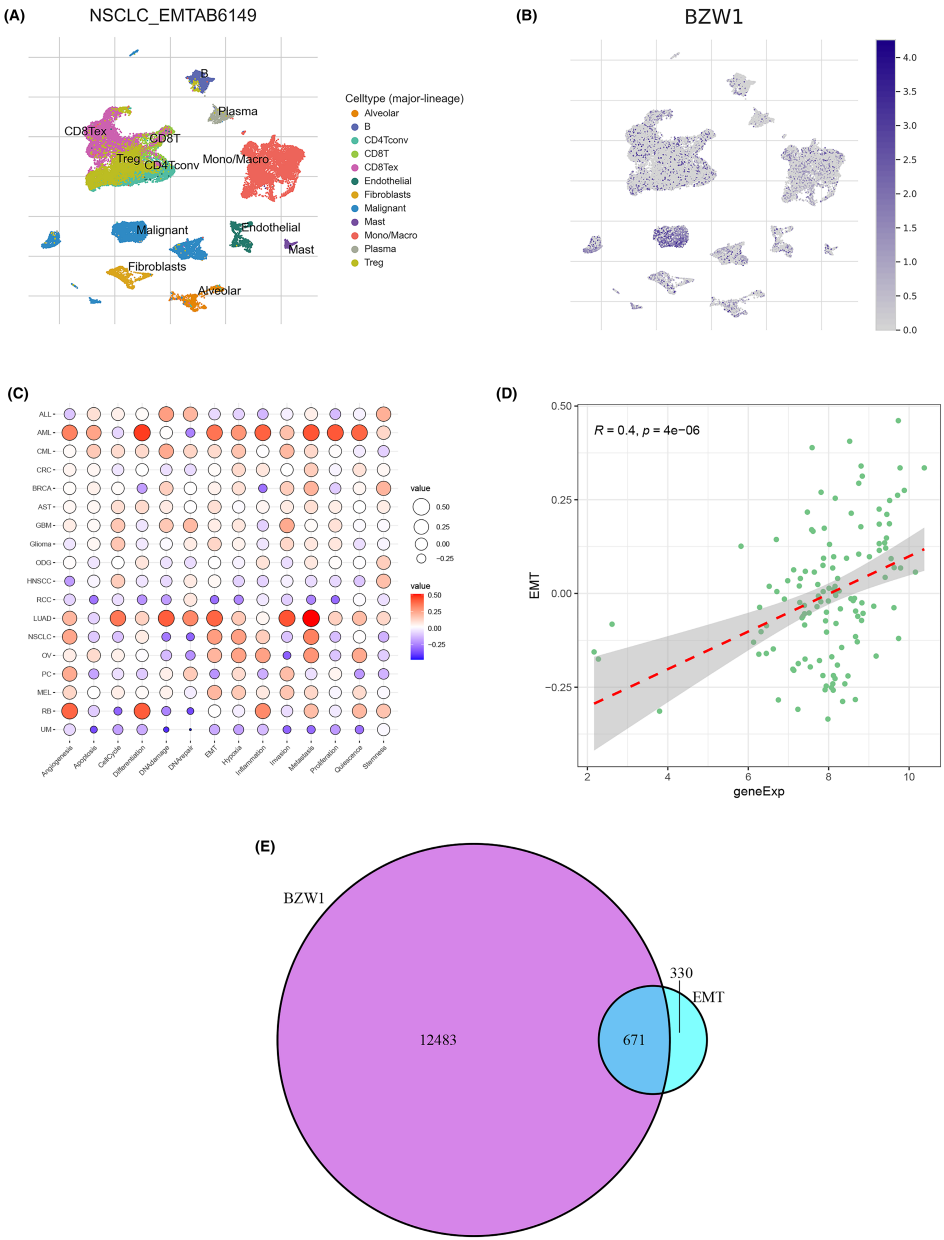
Single‐Cell Sequencing Analysis of Tumour‐Associated Processes. (A, B) Cell classification and annotation results. (C) Correlation of BZW1 with tumour‐associated processes. (D) Quantified correlation between BZW1 and the EMT process. (E) Venn diagram showing the intersection genes between BZW1‐related genes and EMT‐related genes.

In further elucidating the cellular functions of BZW1, we conducted additional analysis utilizing single‐cell data from the GEO database of LUAD. Our analysis of cell annotation results revealed a significant elevation of BZW1 expression in malignant tumour cells, consistent with our prior findings (Figure 3A). Additionally, we observed heightened expression of BZW1 in immune cells, while its abundance in fibroblasts exceeded that in epithelial cells (Figure 3B). To corroborate our conclusions, we performed an analysis of tumour‐related immune processes using single‐cell data from CancerSEA, revealing close associations between BZW1 and EMT (Figure 3C). This relationship was further visualized in Figure 3D. Moreover, we found that BZW1 is associated with tumour cell behaviours, including cell invasion, cell cycle and tumour metabolism.

### 3.4 The correlation between BZW1 and cellular migration

**FIGURE 4:**
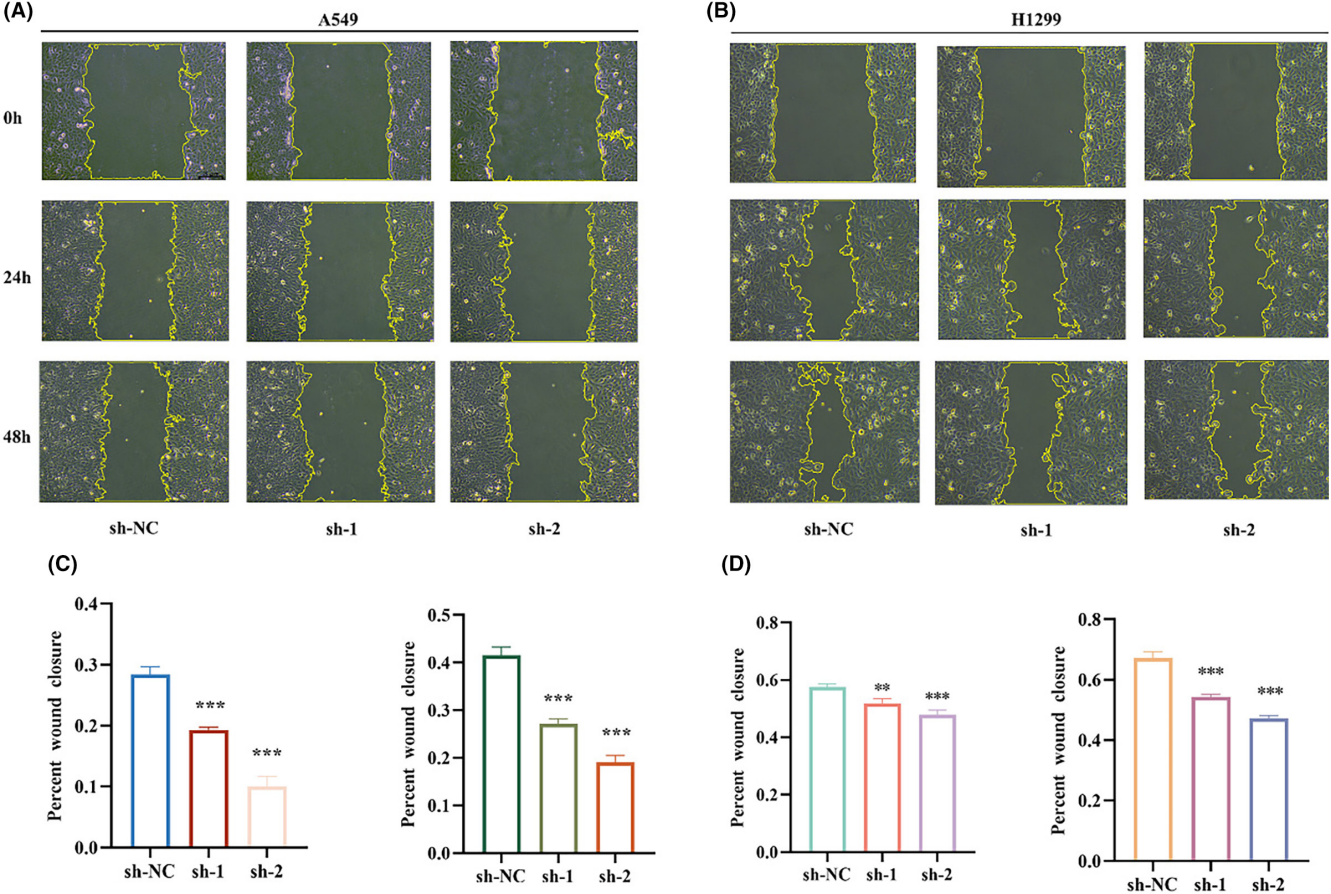
Scratch Assay of A549 and H1299 Cells. (A, C) Scratch assay of A549 cells at 0 h, 24 h and 48 h after BZW1 knockdown (*n* = 5) (Scar bar = 25 μm). (B, D) Scratch assay of H1299 cells at 0, 24 and 48 h after BZW1 knockdown (n = 5) (Scar bar = 25 μm). (**p < 0.01, ***p < 0.001).

**FIGURE 5:**
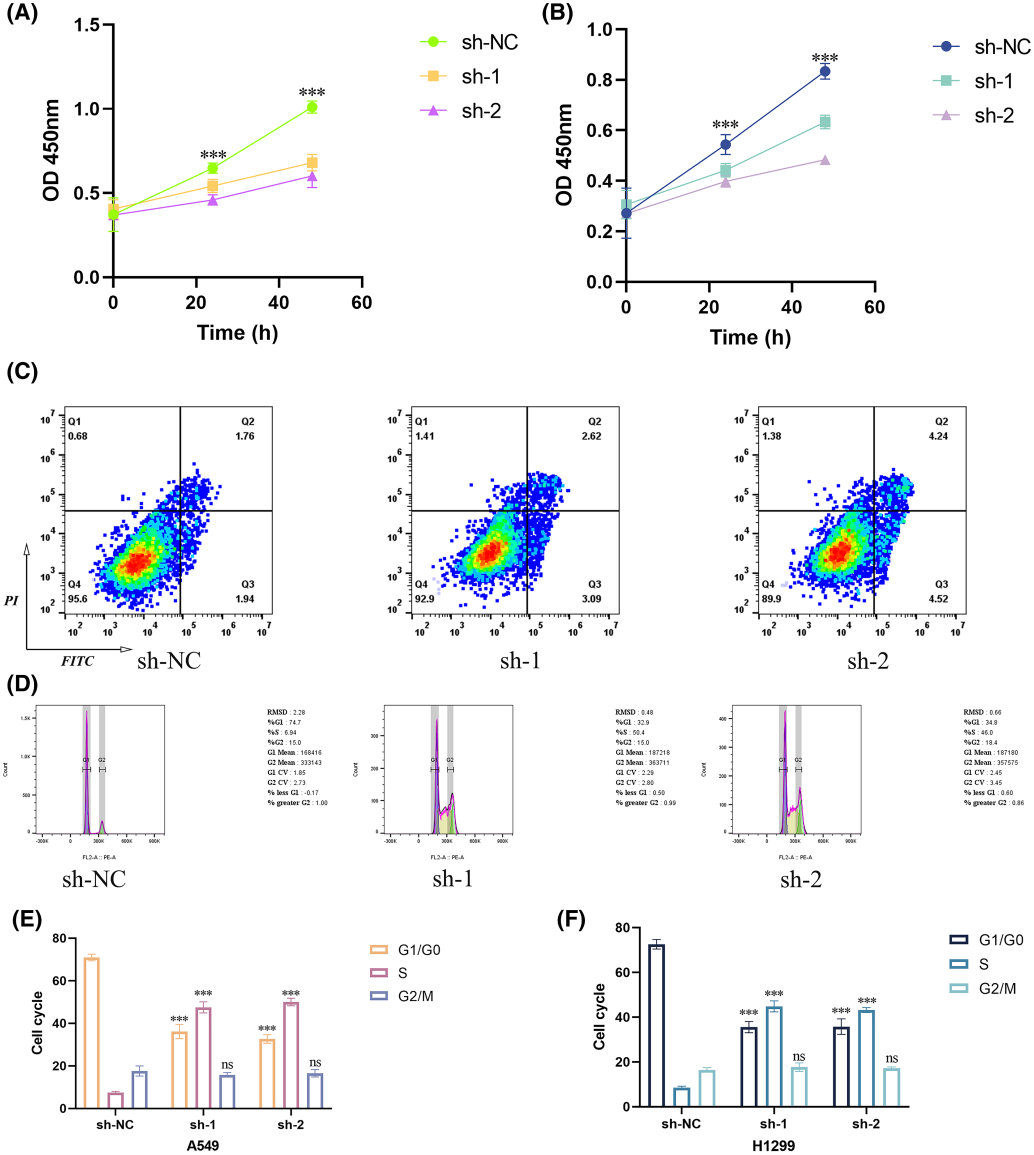
Correlation of BZW1 with apoptosis and cell cycle. (A) CCK8 assay in A549 cells. (B) CCK8 assay in H1299 cells. (C) Apoptosis assay in A549 cells. (D) Cell cycle assay in A549 cells. (E) Quantitative relationship graph of the cell cycle in A549 cells. (F) Quantitative relationship graph of cell cycle in H1299 cells. ***p < 0.001).

To validate the reliability of the screened tumour‐related functions of BZW1 in the bioinformatics database, we conducted subsequent experiments using two commonly used LUAD cell lines, A549 and H1299. We found that after knocking down BZW1, the migration ability of A549 cells significantly decreased (Figure 4A,C). This phenomenon was also validated in H1299 cells (Figure 4B,D). To further confirm this observation, we performed CCK8 experiments to detect cell viability and the results showed a significant decrease in cell viability after knocking down BZW1 (Figure 5A,B). Therefore, high expression of BZW1 promotes the invasive activity of tumour cells.

### 3.5 The impact of bzw1 on cellular apoptosis and cell cycle regulation

To validate the accuracy of single‐cell sequencing results, we conducted further experiments using FITC and PI dyes. Figure 5C and Figure S1A illustrate the apoptosis rates of two cell types upon BZW1 knockdown, revealing an increase in apoptosis following BZW1 downregulation. Additionally, cell cycle experiments demonstrated a stalling of cell cycle progression upon inhibition of BZW1 expression. Figure 5D and Figure S2 depict alterations in the cell cycle of the two lung adenocarcinoma cell lines after BZW1 reduction. Figure 5E,F provide quantitative analysis of the cell cycle process in A549 and H1299 cells, respectively.

### 3.6 Exploring the link between BZW1 and EMT

**FIGURE 6:**
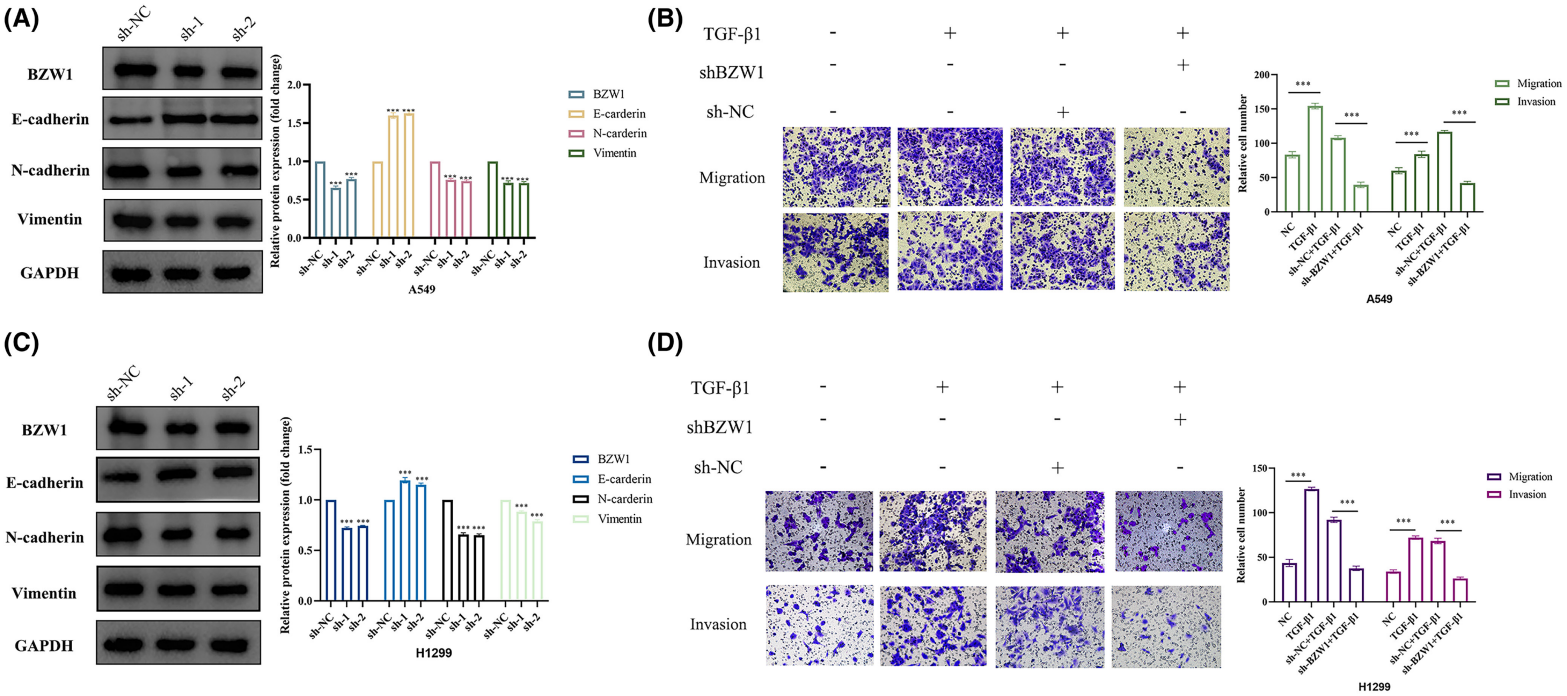
Correlation Between BZW1 and EMT. (A) Correlation between BZW1 and EMT in A549 cells. (B) Involvement of BZW1 in TGF‐β1‐induced EMT process in A549 cells (Scar bar = 50 μm). (Scar bar = 50 μm) (C) Correlation between BZW1 and EMT in H1299 cells. (D) Involvement of BZW1 in TGF‐β1‐induced EMT process in H1299 cells (Scar bar = 50 μm). (***p < 0.001).

In order to investigate the relationship between BZW1 and EMT, we conducted follow‐up experiments. Upon BZW1 knockdown, we observed a significant increase in the epithelial marker and a noticeable decrease in the mesenchymal markers N‐cadherin and Vimentin (Figure 6A,C). Furthermore, to further elucidate the impact of BZW1 on EMT, we supplemented with it the EMT‐inducing growth factor TGF‐β1. Post supplementation, we observed a significant increase in migration and invasion of A549 and H1299 cells compared to the control group. However, this phenomenon was reversed upon BZW1 knockdown (Figure 6B,D). In summary, our analysis suggests that elevated expression of BZW1 promotes EMT in tumour cells, thereby enhancing their invasion and migration capabilities.

### 3.7 BZW1 involvement in influencing the EMT process of LUAD via the Wnt/β‐catenin pathway

**FIGURE 7:**
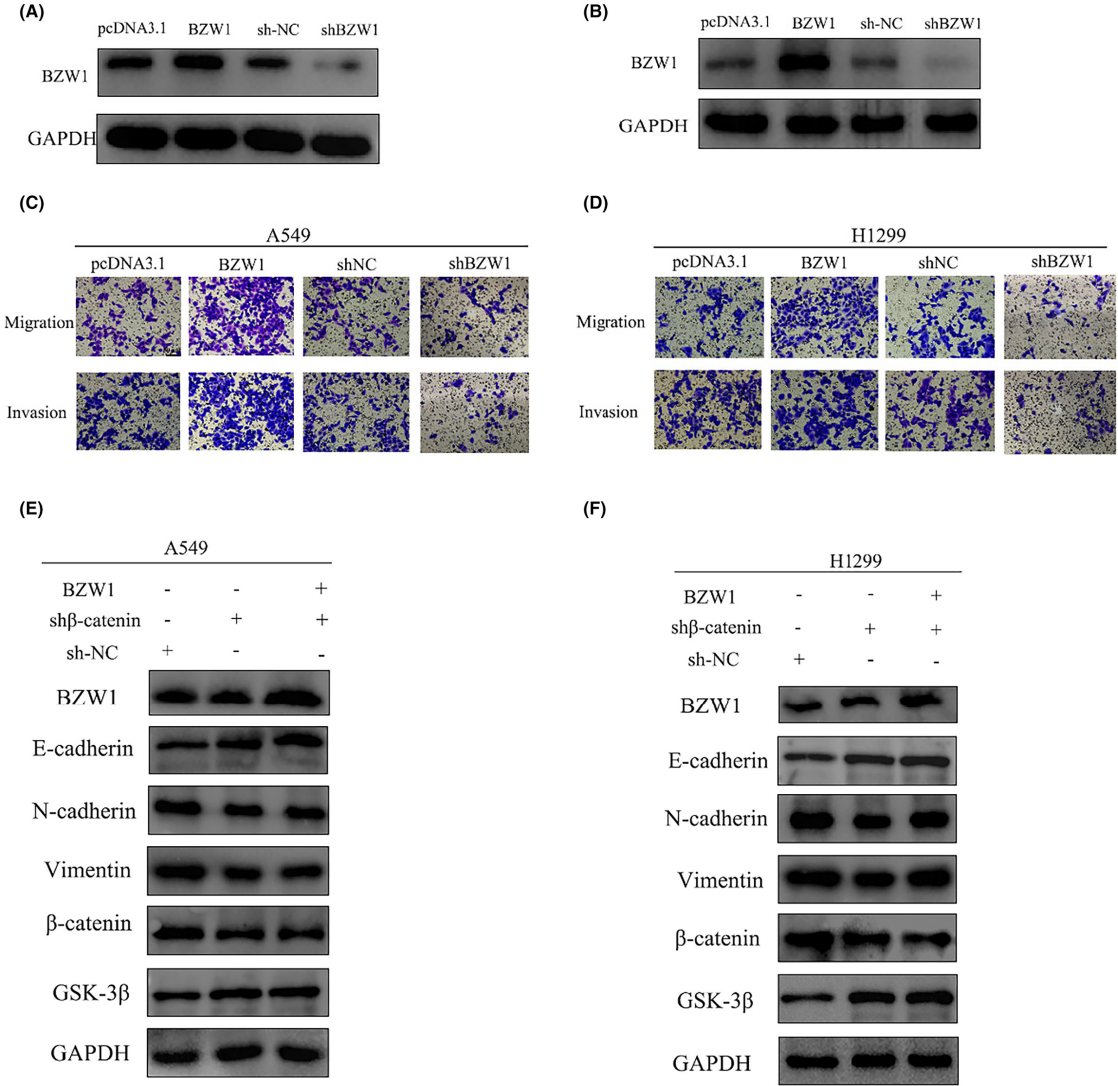
Impact of BZW1 knockdown and overexpression on EMT. (A) Western blot analysis of BZW1 knockdown and overexpression efficiency in A549 cells. (B) Western blot analysis of BZW1 knockdown and overexpression efficiency in H1299 cells. (Scar bar&amp;#x02009;=&amp;#x02009;50&amp;#x02009;μm). (C) Effect of BZW1 knockdown and overexpression on tumour cell migration and invasion in A549 cells. (D) Effect of BZW1 knockdown and overexpression on tumour cell migration and invasion in H1299 cells. (Scar bar&amp;#x02009;=&amp;#x02009;50&amp;#x02009;μm). (E) Expression levels of EMT key proteins and Wnt pathway components upon blockade of the Wnt key protein β‐catenin in A549 cells. (F) Expression levels of EMT key proteins and Wnt pathway components upon blockade of the Wnt key protein β‐catenin in H1299 cells.

Through transfection of plasmids encoding slow viruses and pcDNA3.1, we successfully established both high and low‐expression groups of BZW1 (Figure 7A,B). Results revealed a significant enhancement in cell migration and invasion capabilities in the high‐expression BZW1 group. In contrast, a notable decrease was observed in these abilities in the low‐expression BZW1 group (Figure 7C,D). β‐catenin protein, a core component of the Wnt pathway, has the ability to activate a cascade of downstream genes leading to alterations. Upon transfection of low expression β‐catenin plasmids, we observed an elevation in the epithelial marker E‐cadherin, while the mesenchymal markers N‐cadherin and Vimentin decreased. This phenomenon persists even in the high‐expression BZW1 group (Figure 7E,F).

**FIGURE 8:**
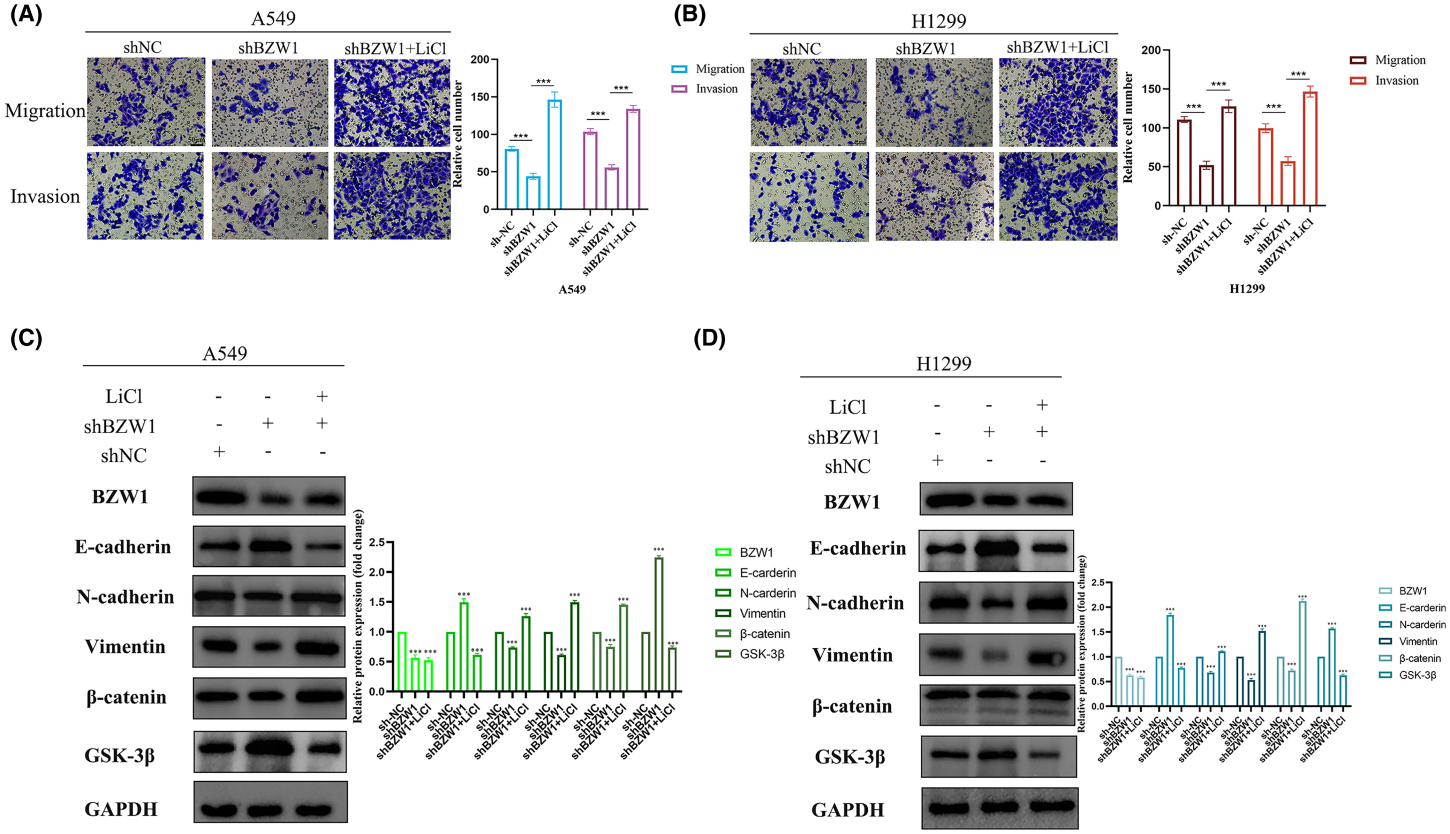
Impact of LiCl pathway activator on BZW1‐induced EMT in lung adenocarcinoma. (A) Effect of adding Wnt pathway activator on simultaneous knockdown of BZW1 on tumour cell invasion and migration capabilities in A549 cells. (Scar bar = 50 μm) (B) Effect of adding Wnt pathway activator on simultaneous knockdown of BZW1 on tumour cell invasion and migration capabilities in H1299 cells. (Scar bar = 50 μm) (C) Western blot analysis of EMT markers and critical proteins in the Wnt pathway in A549 cells. (D) Western blot analysis of EMT markers and critical proteins in the Wnt pathway in H1299 cells (***p < 0.001).

To further illustrate that BZW1 can influence tumour cell EMT through the Wnt/β‐catenin pathway, we conducted additional experiments using the Wnt/β‐catenin pathway activator LiCl. Results showed that upon knocking down BZW1, cell migration and invasion significantly decreased; however, this effect was reversed upon the addition of the Wnt pathway activator (Figure 8A,B). Additionally, protein detection results revealed that upon knocking down BZW1 expression, the Wnt pathway negative regulator GSK‐3β expression increased. However, upon the addition of the Wnt pathway activator LiCl, the epithelial marker E‐cadherin decreased, while the mesenchymal markers N‐cadherin and Vimentin increased (Figure 8C,D). Through the analysis above, we propose that BZW1 participates in the EMT process of LUAD cells via the Wnt/β‐catenin pathway.

### 3.8 Construction of prognostic model for BZW1‐related genes using machine learning algorithms

**FIGURE 9:**
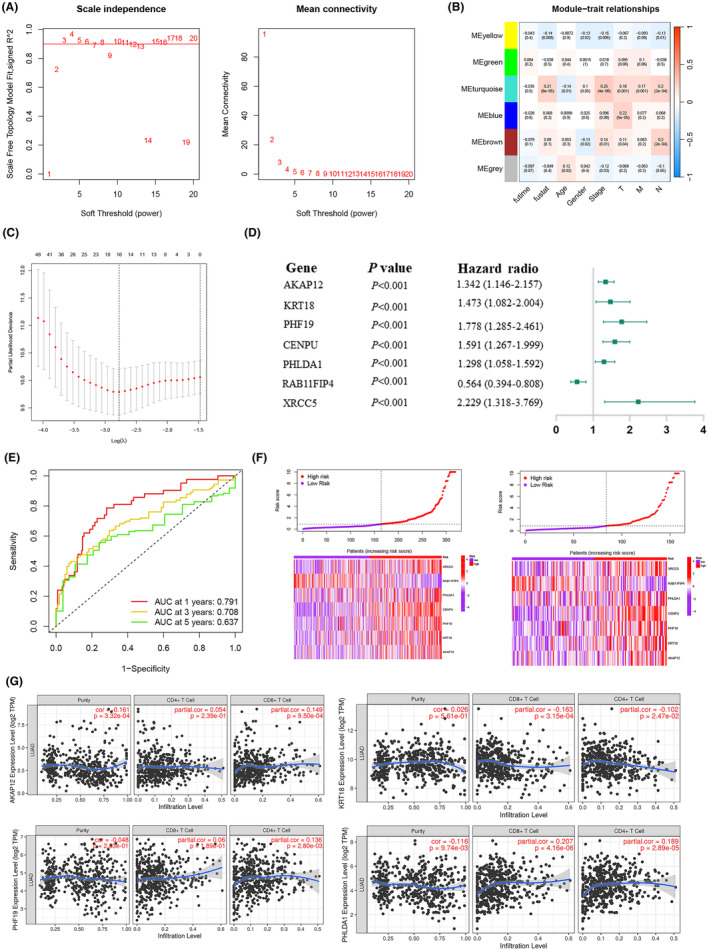
Construction of Prognostic Model for BZW1‐Associated Genes Using Machine Learning. (A, B) Analysis of BZW1‐associated genes and their intersection with EMT genes using the WGCNA algorithm. (C) Further selection of core genes utilizing the LASSO algorithm. (D) Cox regression analysis of genes within the prognostic model. (E) Area under the ROC curve of the prognostic model. (F) The risk curve of the prognostic model. (G) Analysis of immune cell correlation of core genes using the Timer database.

Through the bioinformatics mentioned above analyses and experimental validation, we have demonstrated that BZW1 can influence the EMT process in LUAD cells via the Wnt/β‐catenin pathway, thereby regulating tumour cell proliferation and migration activities. To further guide clinical interventions, we intersected BZW1‐associated genes with EMT genes (Figure 3E). Utilizing the WGCNA algorithm, we identified genes most relevant to patient prognosis, highlighting the association with tumour stage and the turquoise module (Figure 9A,B). Employing LASSO regression on genes within the turquoise module, we identified seven core genes (Figure 9C). Multifactorial Cox values and P values of these seven core genes are depicted in Figure 9D. Additionally, we observed significant significance in the ROC curve of the prognostic model constructed using these seven core genes, as demonstrated in Figure 9E and Figure S3, showcasing clear discrimination between high and low‐risk scores (Figure 9F). Furthermore, considering the pivotal role of immune cells in patient prognosis, we analysed the immune cell correlation of the seven genes using the Timer2.0 database, with results presented in Figure 9G.

### 3.9 Immune cell infiltration analysis of BZW1

**FIGURE 10:**
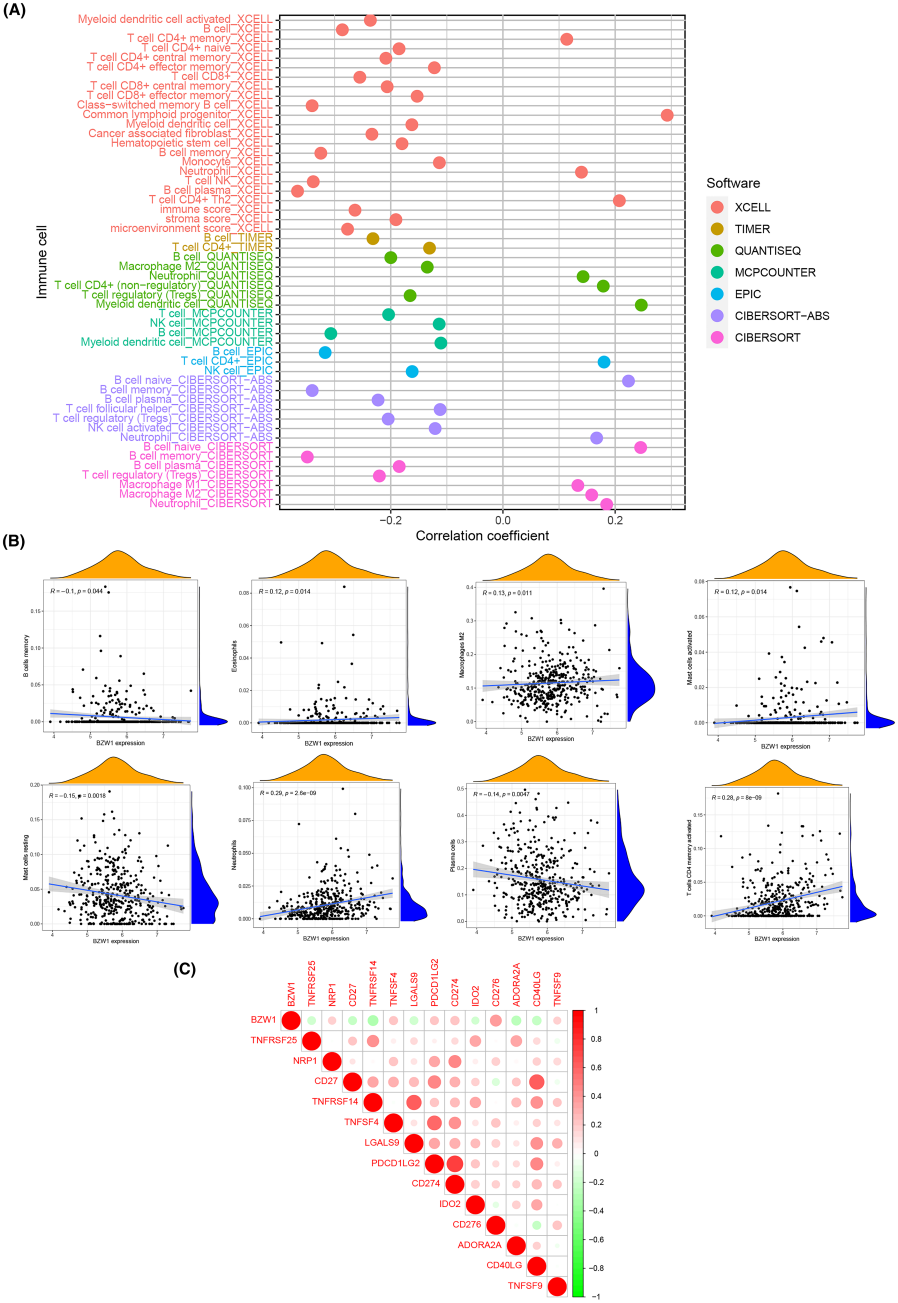
Immunological Cell Infiltration Analysis of BZW (A) Results of Immunological Cell Infiltration Analysis of BZW1 from Multiple Databases (B) Correlation Analysis of BZW1 with Memory B Cells, Eosinophils, M2 Macrophages, Activated Mast Cells, Neutrophils, Plasma Cells and Activated Memory CD4+ T Cells (C) Correlation between BZW1 and Immune Checkpoints.

Immunocellular dynamics play a pivotal role in tumour initiation and progression. Thus, we conducted an immune infiltration analysis focusing on BZW1. Leveraging various databases, including Timer, XCELL, QUANTISEQ, MCPCOUNTER, CIBERSORT and EPIC, we observed a significant correlation between BZW1 and regulatory T cells as well as quiescent B cells in LUAD (Figure 10A). Additionally, we further visualized immune cell infiltration results from CIBERSORT for enhanced visualization, as depicted in Figure 10B. Moreover, considering the crucial role of immune checkpoints in tumorigenesis, we explored the correlation between BZW1 and immune checkpoints. Our findings revealed a prominent positive correlation between BZW1 and CD276, while a notable negative correlation was observed with TNFRSF14 (Figure 10B). Immune cells are pivotal in tumour initiation and progression. Thus, our study provides insights into BZW1‐associated immune cell dynamics and its relevance to cancer immunotherapy (Figure 10C).

## 4 DISCUSSION

In this article, we systematically analysed the role of BZW1 in cancer. Utilizing various databases, we conducted a pan‐cancer analysis of BZW1 and observed its overexpression across multiple cancer types. Furthermore, leveraging single‐cell sequencing data from LUAD, we identified heightened expression of BZW1 in both fibroblasts and malignant cells. To further validate this phenomenon, we delved into single‐cell data from the CancerSEA database, revealing a close association between BZW1 and the tumour‐related process of EMT. Subsequently, to elucidate this phenomenon, we conducted experiments across multiple cell lines. Results showed that BZW1 significantly influenced cell viability, proliferation, invasion and cell cycle‐related processes. Moreover, we elucidated that BZW1 modulates the EMT process in LUAD via the Wnt/β‐catenin signalling pathway. In guiding clinical interventions, we intersected EMT genes with BZW1‐related genes and successfully constructed a prognostic model using various machine‐learning algorithms, demonstrating robust predictive capabilities.

As a recently discovered cancer gene, BZW1 has been the subject of limited research.^7^ With the rise of single‐cell sequencing technologies in recent years, many phenomena previously masked by widespread sequencing have gradually been uncovered. Li et al. successfully demonstrated that BZW1 can promote glycolysis in pancreatic ductal adenocarcinoma by enhancing eIF2α, consistent with our single‐cell sequencing analysis showing that BZW1 affects metabolism and thus tumour growth outcomes.^5^, ^21^ Furthermore, BZW1 has been shown to influence tumour cell development by affecting various signalling pathways. Researchers found that BZW1 promotes cell proliferation by modulating the TGF‐β1/Smad pathway.^22^ In glioma, the lncRNA NEAT1 promotes tumour progression by regulating the miR‐98‐5p/BZW1 axis.^23^

Wnt, one of the world's oldest signalling pathways, has been shown to play a role in various cancers.^24^ The methods of immunotherapy and targeted therapy for lung cancer have gradually increased, with the use of inhibitors showing potential in blocking the occurrence and progression of the disease.^25^, ^26^ BZW1, as a novel cancer gene, may play a crucial role in the treatment of lung cancer when targeted with specific inhibitors. Recent studies have found that tumour genes can regulate lung cancer cell epithelial‐mesenchymal transition (EMT) by affecting the Wnt signalling pathway, thereby controlling tumour growth. Our research similarly demonstrates that BZW1 can influence tumour cell proliferation by modulating the Wnt/β‐catenin signalling pathway, consistent with previous studies.^27^ However, our research indicates that reducing BZW1 levels promotes tumour cell apoptosis, contradicting database results. Nevertheless, through experimentation, we have shown that reducing BZW1 significantly inhibits cell activity and cell cycle progression. Therefore, we still believe that reducing BZW1 promotes tumour cell apoptosis. This discrepancy may stem from insufficient sequencing depth or quantity. In recent years, with the increasing application of machine learning algorithms in medicine, many articles have been utilizing single‐gene constructs to build prognostic models and survival curves. Similarly, we have successfully constructed a prognostic model using BZW1‐related genes and its tumour cell process. The area under the ROC curve (AUC) successfully validates the accuracy of this prognostic model and its clinical value.

## 5 CONCLUSION

In recent years, the discovery of BZW1 as a novel cancer gene holds significant scientific significance. Utilizing extensive single‐cell sequencing data analysis, we delineated the potential impact of BZW1 on various tumour‐related processes. Furthermore, experimental validation underscored BZW1's capacity to influence tumour EMT through modulation of the Wnt/β‐catenin pathway. Additionally, leveraging EMT, we successfully devised a prognostic model centered on BZW1. These findings not only expand our understanding of BZW1's role but also provide a robust foundation for further research and clinical applications.

## AUTHOR CONTRIBUTIONS

**Wei Lai**:Conceptualization (lead); resources (equal); software (equal); supervision (equal); validation (equal); original draft (equal); writing—review and editing (equal) **Zhou Ping**: Conceptualization (equal); resources (equal); software (equal); supervision (equal); **Yun Chen**: Original draft (equal); writing—review and editing (equal); **Junrong Wang:** Software (equal); **Yuyan Liiu**: Writing—review (equal); **Shishi Zou**: Resources (equal); **Jieweng Wang**: Editing (equal); **Tianyu Zhang**: Editing (equal) **Wei Ren**: Writing—review and editing (equal); **Wei Wang**: Original draft (equal); writing—review and editing (equal).

## FUNDING INFORMATION

This work was supported by the National Natural Science Foundation of China (82300539) and Natural Science Foundation of Hubei (2022CFB295).

## CONFLICT OF INTEREST STATEMENT

The authors declared that we have no financial or non‐financial conflicts of interest regarding the content of this manuscript.

## CONSENT

Not Applicable.

## Supporting information


Figure S1.

## Data Availability

Public data URLs are described in the text and the data are open for download and use. Experimental raw data are available through the corresponding author.
