# Supplementary material for: Multi‐omics analysis reveals BZW1's regulation of EMT via the Wnt pathway in lung adenocarcinoma
Source: J Cell Mol Med. 2024 Oct 27;28(20):e70163. doi: 10.1111/jcmm.70163 (PMC11512756; doi:10.1111/jcmm.70163)
Supplement: Supplementary file 1 — Figure S1. [file JCMM-28-e70163-s001.docx]

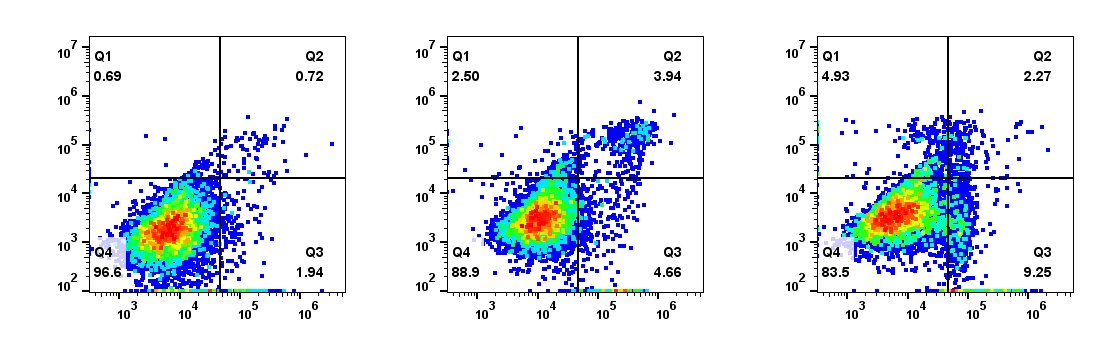


sh-NC sh-1 sh-2

**Supplement figure 1:** H1299 apoptosis


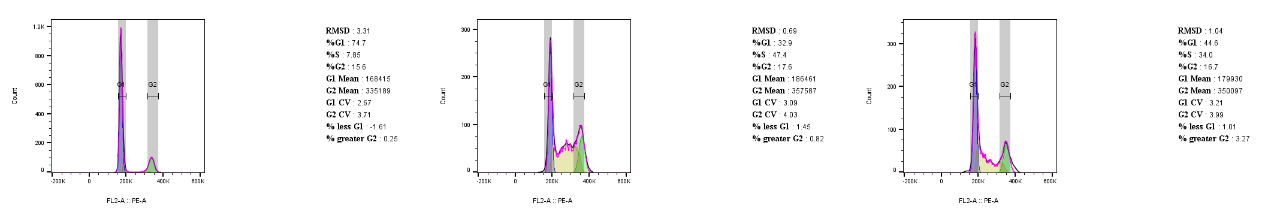


sh-NC sh-1 sh-2

**Supplement figure 2:** H1299 cell cycle


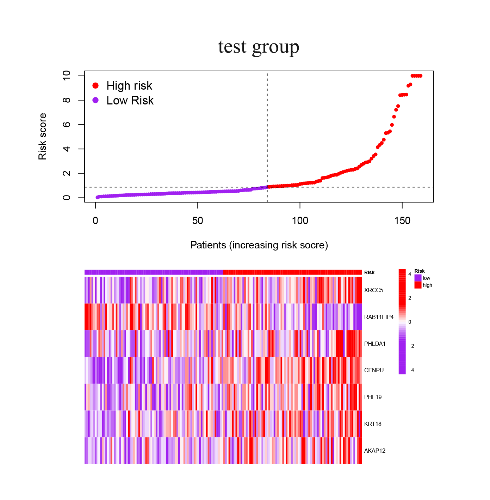


**Supplement figure 3:** test group survival curve
